# Identification of *floR* Variants Associated With a Novel Tn*4371*-Like Integrative and Conjugative Element in Clinical *Pseudomonas aeruginosa* Isolates

**DOI:** 10.3389/fcimb.2021.685068

**Published:** 2021-06-21

**Authors:** Changrui Qian, Hongmao Liu, Jiawei Cao, Yongan Ji, Wei Lu, Junwan Lu, Aifang Li, Xinyi Zhu, Kai Shen, Haili Xu, Qianqian Chen, Wangxiao Zhou, Hongyun Lu, Hailong Lin, Xueya Zhang, Qiaoling Li, Xi Lin, Kewei Li, Teng Xu, Mei Zhu, Qiyu Bao, Hailin Zhang

**Affiliations:** ^1^ The Second Affiliated Hospital and Yuying Children’s Hospital, Wenzhou Medical University, Wenzhou, China; ^2^ Key Laboratory of Medical Genetics of Zhejiang Province, Key Laboratory of Laboratory Medicine, Ministry of Education, China, School of Laboratory Medicine and Life Sciences, Wenzhou Medical University, Wenzhou, China; ^3^ Institute of Biomedical Informatics, School of Laboratory Medicine and Life Sciences, Wenzhou Medical University, Wenzhou, China; ^4^ The Fifth Affiliated Hospital of Wenzhou Medical University, Lishui, China; ^5^ The Second People’s Hospital of Tongxiang City, Tongxiang, China; ^6^ Institute of Translational Medicine, Baotou Central Hospital, Baotou, China; ^7^ Tongji University School of Medicine, Shanghai, China; ^8^ Department of Clinical Laboratory, Zhejiang Hospital, Hangzhou, China

**Keywords:** *floR*, florfenicol resistance, *Pseudomonas aeruginosa*, Tn*4371*, integrative and conjugative elements

## Abstract

Florfenicol is widely used to control respiratory diseases and intestinal infections in food animals. However, there are increasing reports about florfenicol resistance of various clinical pathogens. *floR* is a key resistance gene that mediates resistance to florfenicol and could spread among different bacteria. Here, we investigated the prevalence of *floR* in 430 *Pseudomonas aeruginosa* isolates from human clinical samples and identified three types of *floR* genes (designated *floR*, *floR-T1* and *floR-T2*) in these isolates, with *floR-T1* the most prevalent (5.3%, 23/430). FloR-T2 was a novel *floR* variant identified in this study, and exhibited less identity with other FloR proteins than FloRv. Moreover, *floR-T1* and *floR-T2* identified in *P. aeruginosa* strain TL1285 were functionally active and located on multi-drug resistance region of a novel incomplete Tn*4371*-like integrative and conjugative elements (ICE) in the chromosome. The expression of the two *floR* variants could be induced by florfenicol or chloramphenicol. These results indicated that the two *floR* variants played an essential role in the host’s resistance to amphenicol and the spreading of these *floR* variants might be related with the Tn*4371* family ICE.

## Introduction

Florfenicol is a fluorinated synthetic analog of thiamphenicol (Syriopoulou et al., 1981), mainly used to control respiratory tract diseases and enteric infections in food-producing animals (Zhao et al., 2016). However, due to inappropriate medication use, florfenicol resistance has become increasingly serious in veterinary medicine (Chang et al., 2014). Although florfenicol is not approved for use in humans, an increasing number of studies have reported dramatic and serious florfenicol resistance in various clinical strains, such as *Pasteurella multocida*, *Salmonella*, and *Klebsiella pneumoniae* (Lu et al., 2018; Ujvari et al., 2019; Zhan et al., 2019).

To date, seven florfenicol resistance genes (excluding variants), *floR*, *fexA*, *fexB*, *cfr*, *optrA, pexA* and *estDL136*, have been reported (Arcangioli et al., 1999; Schwarz et al., 2000; Kehrenberg and Schwarz, 2004; Schwarz et al., 2004; Lang et al., 2010; Liu et al., 2012; Tao et al., 2012; Wang et al., 2015). Among them, *floR* is one of the main florfenicol resistance genes in Gram-negative bacteria (He et al., 2015). Several variants of the *floR* gene, including *pp-flo*, *cmlA*-like, *floRv* and *floSt*, have been documented, and most of them encode 404 aa proteins. These *floR* variants are closely related to each other, and *floRv* from *Stenotrophomonas maltophilia* shares the lowest amino acid identity (88.4%-91.8%) with the others excluding *pp-flo* (He et al., 2015). The *floR* gene has been identified either on chromosomes or plasmids of various bacteria and has often been associated with mobile genetic elements and genomic islands (Lai et al., 2013; Gabida et al., 2015; da Silva et al., 2017).

*Pseudomonas aeruginosa* is an opportunistic pathogen that can cause numerous acute or chronic infections, and is notorious for its intrinsic and acquired resistance to numerous antibiotics (Breidenstein et al., 2011; Domalaon et al., 2018). Generally, *P. aeruginosa* chromosomes do not carry the *floR* gene. Although *P. aeruginosa* is clinically resistant to chloramphenicol (Morita et al., 2014), rifampicin-tobramycin conjugates could break the intrinsic resistance of *P. aeruginosa* to chloramphenicol *in vitro* and *in vivo*, making it suitable for clinical treatment (Idowu et al., 2019). However, the *floR* gene carried by this pathogen may cause this strategy to fail when chloramphenicol is used. The prevalence of the *floR* gene in *P. aeruginosa* hasn’t been previously investigated. In this study, we determined the prevalence of *floR* gene among 430 clinical *P. aeruginosa* isolates collected from Wenzhou, China in the years 2008-2009 and 2015-2017. The combination of whole-genome sequencing, genotyping and gene expression methods was used to characterize the *floR* variants. A novel Tn*4371*-like integrative and conjugative element (ICE) carrying *floR-T1* and *floR-T2* was identified, which indicated that the Tn*4371*-like ICE might play an important role in the dissemination of *floR-T2*.

## Materials and Methods

### Bacterial Isolation

A total of 430 clinical *P. aeruginosa* strains isolated from sputum, urine or blood samples of patients were collected from a teaching hospital of Wenzhou Medical University. Among these isolates, 200 strains were isolated during 2008-2009, and 230 strains were isolated in 2015-2017. The strains were identified using the Vitek-60 microorganism auto-analysis system (BioMerieux Corporate, Craponne, France).

### Antimicrobial Susceptibility Testing

Minimum inhibitory concentrations (MICs) of 17 antimicrobial agents were determined using an agar dilution method with Mueller-Hinton agar recommended by the Clinical and Laboratory Standards Institute (CLSI document M100-S27, 2017). Broad range concentrations of 0.125-1024 μg/mL were used for all the agents. MICs were interpreted according to CLSI breakpoints for *P. aeruginosa*.

### DNA Extraction and Sequencing

Each purified isolate was incubated overnight in 5 ml of Luria-Bertani (LB) broth at 37°C for 16 hours, and genomic DNA was extracted using an AxyPrep Bacterial Genomic DNA Miniprep kit (Axygen Scientific, Union City, CA, USA). According to the time period of isolation, two mixed DNA collections consisting of equal amounts of genomic DNA of each strain were obtained. One collection (designated TL0809) contained the bacteria isolated from 2008-2009 and the other (designated TL151617) contained those isolated among 2015-2017. The library with an average insert size of 400 bp was prepared using NEBNext Ultra II DNA library preparation kit, and subsequently high-throughput sequenced by the Illumina Novaseq (paired-end run; 2×150 bp). In addition, a 10- to 20-kb insert library was obtained from the genomic DNA of *P. aeruginosa* TL1285 and sequenced by Pacific Bioscience RSII sequencers at Annoroad Gene Technology Co., Ltd. (Beijing, China).

### Genome Assembly, Annotation, and Bioinformatics Analysis

Genome assembly of pooled DNA sequencing data was performed using megahit (Li et al., 2015), and contigs less than 400 bp were discarded. The complete genome of *P. aeruginosa* TL1285 was assembled using Canu (Koren et al., 2017) with long reads obtained from PacBio sequencing. Error correction of tentative complete circular sequence was performed using Pilon (Walker et al., 2014) with short read sets derived from Illumina sequencing. Open reading frames (ORFs) of pooled DNA sequences were predicted using Prodigal (Hyatt et al., 2010) with default parameters. Using the antibiotic resistance genes of the CARD (Jia et al., 2017) and ResFinder (Ea et al., 2012) databases as a query, a BLASTN search was performed against the two assembled sequences of the pooled DNA with thresholds of >70% nucleotide identity and >80% alignment coverage. Gene prediction and annotation of TL1285 were initially performed with RAST (Aziz et al., 2008) and then verified by BLASTP searches against the UniProtKB/Swiss-Prot (Boutet et al., 2016) and RefSeq (O’Leary et al., 2016) databases. Annotation of mobile genetic elements was carried out using online databases including ISfinder (Siguier et al., 2006), INTEGRALL (Moura et al., 2009), and the Tn Number Registry (Roberts et al., 2008). Comparison of the TL1285 genome with the other six genomes was performed using BLAST Ring Image Generator (Alikhan et al., 2011). Gene organization diagrams were generated using R script and modified with Inkscape 1.0 (https://inkscape.org/en/).

### PCR Amplification and Cloning of the *floR* Gene

Genomic DNA of each of the 430 isolates was screened for the *floR* gene using PCR with primers listed in **Table 1**. PCR amplification was carried out under the following conditions: initial denaturation for 10 min at 94°C; 35 cycles of denaturation (30 s at 94°C), annealing (30 s at 58°C) and extension (90 s at 72°C) and a final extension for 10 min at 72°C. The *floR-T1* and *floR-T2* gene sequences with promoter regions were amplified from *P. aeruginosa* TL1285 and cloned into pUCP24. Electroporation transformation was used to introduce the recombinant plasmids into *P. aeruginosa* PAO1 by Bio-rad MicroPulser with a voltage at 2.6 kv, resistance at 200 Ω and pulse time of 5 ms (Dennis and Sokol, 1995).

**Table 1:** PCR primers used in this study.

Primers	Purposes	Sequences	Product size (bp)
s-*floR*-F	screening of *floR*	GCGCAACGGCTTTCGTCATT	270
s-*floR*-R		GCATCGCCAGTATAGCCAAA	
s-*floR-T1*-F	screening of *floR-T1*	GCGCAACGGCTTTCGTTGCT	262
s-*floR-T1*-R		GCGAAGCCAGTGCAGCCAGT	
s-*floR-T2*-F	screening of *floR-T2*	GGGCCATACTTTTCATCGTC	278
s-*floR-T2*-R		TCAACGCCAGCACAGCAAGC	
c-floR-T1-F	cloning of *floR-T1*	GGGATTCGGTGAGAAATGGCTACG	1600
c-*floR-T1*-R		AATGAGCGGTATCTTGCCAGACAG	
c-*floR-T2*-F	cloning of *floR-T2*	AATCCCATGAGTTCACCCTCGTTCC	1500
c-*floR-T2*-R		AATGAGCGGTATTCTGCCGGACAG	
q-*floR-T1*-F	*floR-T1* qRT-PCR	GCGACGTATATGCCAATCGT	184
q-*floR-T1*-R		CTGAAACTGGCGTTTAAGAG	
q-*floR-T2*-F	*floR-T2* qRT-PCR	ATCTTCGCGAGTCCAGCCTT	200
q-*floR-T2*-F		TCTGGCGACAAAGGACTTCG	
PA_16S_ -F	*P. aeruginosa* 16S rRNA qRT-PCR	AACGCGAAGAACCTTACC	149
PA_16S_-R		AAGGGTTGCGCTCGTTAC	
EC_16S_-F	*E. coli* 16S rRNA qRT-PCR	AATGCCACGGTGAATACG	153
EC_16S_-R		CTACGGTTACCTTGTTACGA	
Tn*4371*-P1	circular forms and insertion sites of Tn*4371*	CGAGAGCGTCAAGCTGACCT	
Tn*4371*-P2		GAGCGTGGGACAGCTGCTT	
Tn*4371*-P3		CAAGGATCGGGCCTTGATGT	

### Comparison of the Expression of *floR-T1* and *floR-T2*

Quantitative reverse transcription PCR (qRT-PCR) was used to investigate the expression of the *floR* variants of TL1285 and transformants in the presence or absence of 2 mg/L florfenicol or chloramphenicol. In brief, RNA was extracted from 3 mL of LB broth culture (OD600 = 1) of *P. aeruginosa* TL1285 and the transformants using TRIzol Reagent (Invitrogen, USA) following the manufacturer’s instructions. RNA (1 μg) was used as the template for cDNA synthesis using HiScript II Reverse Transcriptase (Vazyme, Nanjing, China) following the manufacturer’s instructions. qRT-PCR was used to quantify the amount of *floR-T1* and *floR-T2* in cDNAs using ChamQ Universal SYBR qPCR Master Mix (Vazyme, Nanjing, China) following the manufacturer’s instructions with the qPCR primers (**Table 1**).

### Detection of the Extrachromosomal Intermediate

Inverse PCR using the primers beside the *attL* and *attR* sites could be utilized for the rapid identification of the extrachromosomal intermediate of Tn*4371* (Ryan et al., 2009). PCR product was obtained only when integrative and conjugative element (ICE) was excised from the chromosome and circularized. Since no *attL* site was identified in TL1285, we designed two primers (P2 and P3) located beside the integrase genes as the forward primers. PCR amplification was carried out under the following conditions: an initial denaturation of 10 min at 94°C; 33 cycles of denaturation (94°C for 30 s), annealing (62°C for 30 s), and extension (72°C for 90 s); and a final extension step at 72°C for 10 min.

### GenBank Accession Number

The complete chromosome sequence of the *P. aeruginosa* TL1285 (CP053390) has been submitted to NCBI GenBank.

### Ethics Approval

This study uses strains obtained from a teaching hospital of Wenzhou Medical University. It did not require the study to be reviewed or approved by an ethics committee because individual patient data was not involved, and only anonymous clinical residual samples during routine hospital laboratory procedures were used in this study.

## Results

### Florfenicol and Chloramphenicol MICs of the Strains

**Figure 1:**
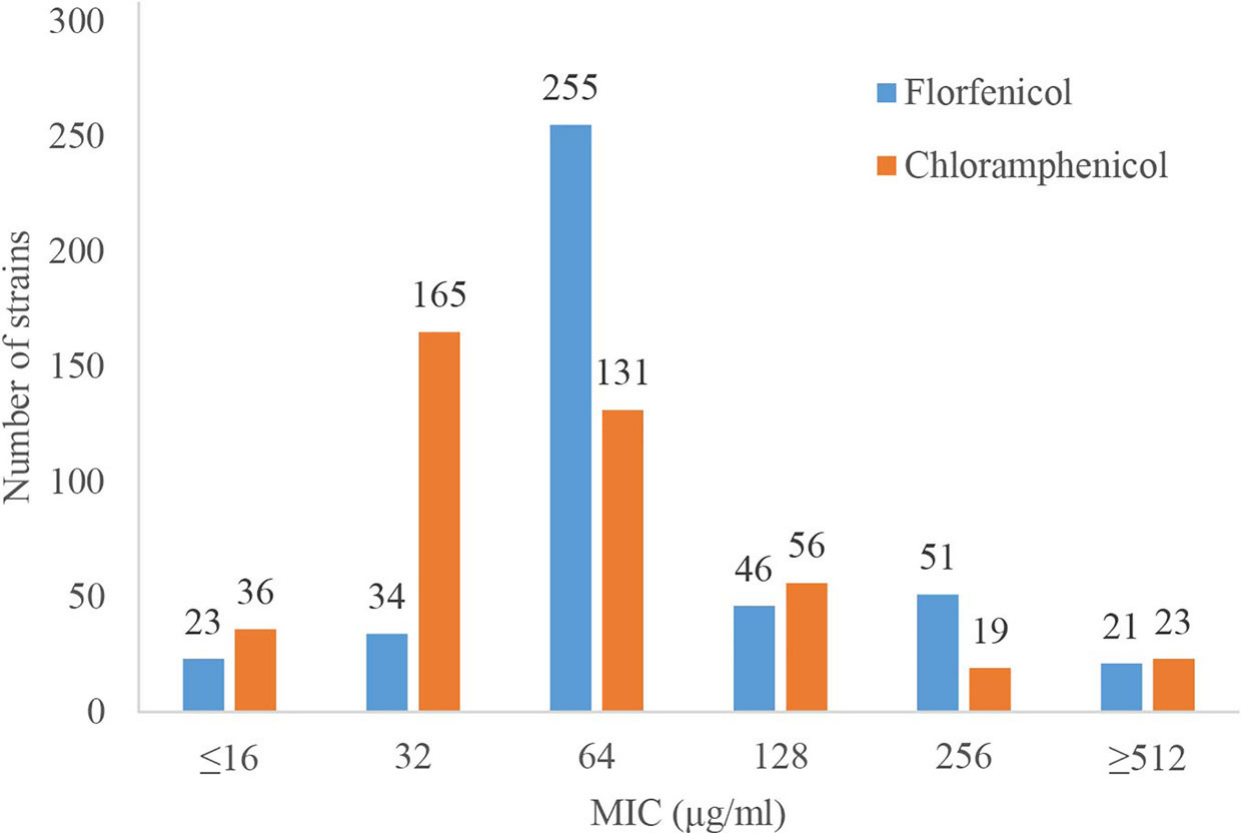
MIC results of the 430 clinical *P. aeruginosa* isolates against florfenicol and chloramphenicol.

The MICs of florfenicol and chloramphenicol were determined for the 430 clinical *P. aeruginosa* isolates. It showed that 21 (4.88%) and 23 (5.35%) of the strains exhibited much higher resistance levels to florfenicol and chloramphenicol with the same MICs of ≥512 μg/mL for them (**Figure 1**). A total of 94.65% (407/430) of the strains were resistant to either florfenicol or chloramphenicol (or both), and only 5.35% (23/430) isolates were susceptible to both florfenicol and chloramphenicol with MIC ≤16 μg/mL.

### Identification of the *floR* Variants

To investigate the prevalence of the *floR* gene among clinical *P. aeruginosa* isolates, two mixed DNA collections TL0809 (containing 200 P*. aeruginosa* strains isolated from 2008-2009) and TL151617 (containing 230 P*. aeruginosa* strains isolated from 2015-2017) were sequenced. Using the *floR* gene (AF231986) as a reference, three types of *floR* variants (the reference *floR, floR-T1* and *floR-T2* in this study) were identified in the two mixed genomes, of which TL0809 contained all three and TL151617 contained only two *floR* variants (the reference *floR* and *floR-T1*) (**Table 2**). Other antimicrobial resistance genes (ARGs) identified in TL0809 and TL151617 were listed in **Table S1**.

**Table 2:** The abundance and PCR positive rates of the *floR* variants.

	*floR* variants	Identity	Match length (aa)	Abundance	PCR positive rate
TL0809	*floR^a^*	99.3%	404	0.68	2/200 (1.00%)
	*floR-T1*	91.3%	404	1.18	6/200 (3.00%)
	*floR-T2*	87.6%	404	0.32	1/200 (0.50%)
TL151617	*floR*	99.3%	404	0.56	3/230 (1.30%)
	*floR-T1*	91.3%	404	4.95	17/230 (7.39%)

a reference floR (AF231986).

The result of PCR amplification of the reference *floR*, *floR-T1* and *floR-T2* genes showed that the most prevalent variant was *floR-T1*, while *floR-T2* was only identified in the isolates collected from 2008-2009 (**Table 2**). The positive rates were consistent with the abundance [expressed as ‘copy of ARG per copy of 16S-rRNA gene’ (Li et al., 2015)] of the corresponding genes in the pooled genomic DNA sequencing libraries. The positive rate of the *floR-T1* gene in the strains collected from 2015-2017 (7.39%, 17/230) was higher compared with that from 2008-2009 (3.00%, 6/200).

### Antimicrobial Susceptibility of *P. aeruginosa* TL1285 and the Recombinants With the Cloned *floR* Variants

Among all *P. aeruginosa* strains, only one strain named TL1285, isolated from a sputum sample in 2008, carried both *floR-T1* and *floR-T2*. *P. aeruginosa* TL1285 was resistant to chloramphenicol, florfenicol and many other antibacterial agents (**Table 3**). The fragment containing *floR-T1* or *floR-T2* gene and its putative promoter region was amplified from TL1285 genomic DNA and subsequently cloned into pUCP24, and then transformed into *E. coli* DH5α and *P. aeruginosa* ΔPAO1 (*P. aeruginosa* PAO1 deleted of *ampG*), respectively. As a result, compared with the recipients (*E. coli* DH5α and *P. aeruginosa* ΔPAO1), the recombinants with the cloned *floR-T1* (DH5α*/*pUCP24-*floR-T1* and ΔPAO1*/*pUCP24-*floR-T1*) increased ≥4 folds of MIC levels to both chloramphenicol and florfenicol and the recombinants with the cloned *floR-T2* (DH5α*/*pUCP24-*floR-T2* and ΔPAO1*/*pUCP24-*floR-T2*) increased ≥8 folds of MIC levels to both chloramphenicol and florfenicol, respectively. The results indicated that the *floR-T1* and *floR-T2* genes of *P. aeruginosa* TL1285 were functionally active.

**Table 3:** MIC results of *P. aeruginosa* TL1285 and recombinants to 17 antibiotics (μg/mL).

Antibiotics	TL1285	DH5α	DH5α/pUCP24-*floR-T1*	DH5α/pUCP24-*floR-T2*	ΔPAO1	ΔPAO1/pUCP24-*floR-T1*	ΔPAO1/pUCP24-floR-T2
Ampicillin	1024	–	–	–	–	–	–
Ceftazidime	<1	–	–	–	–	–	–
Levofloxacin	<0.5	–	–	–	–	–	–
Cefpyridine	4	–	–	–		–	–
Minocycline	64	–	–	–		–	–
Chloramphenicol	128	4	64	64	32	128	512
Florfenicol	256	4	64	128	32	256	>1024
Ciprofloxacin	2	–	–	–		–	–
Azithromycin	32	–	–	–		–	–
Fosfomycin	256	–	–	–		–	–
Tigecycline	4	–	–	–		–	–
Colistin	<1	–	–	–	–	–	–
Erythromycin	256	–	–	–	–	–	–
Nalidixic acid	>1024	–	–	–	–	–	–
Gentamicin	>1024	–	–	–		–	–
Kanamycin	64	–	–	–		–	–
Streptomycin	>1024	–	–	–	–	–	–

### Expression of the *floR* Variants

**Figure 2:**
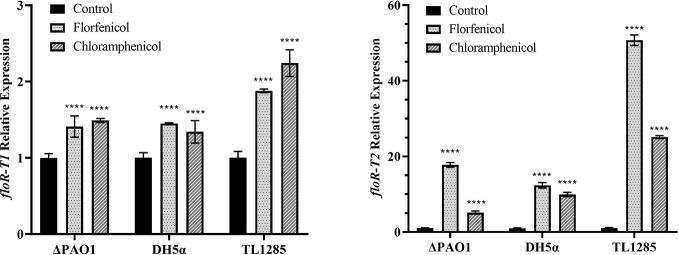
Expression of the *floR* variants in TL1285 and corresponding transformants with or without florfenicol or chloramphenicol induction. **** (P-value < 0.0001).

The expression of the two *floR* variants with or without florfenicol (or chloramphenicol) induction were detected (**Figure 2**). It revealed that the mRNA levels of *floR-T2* in *P. aeruginosa* TL1285 and the corresponding transformants (DH5α*/*pUCP24-*floR-T2* and ΔPAO1*/*pUCP24-*floR-T2*) were significantly increased, while the mRNA levels of *floR-T1* in *P. aeruginosa* TL1285 and the transformants (DH5α*/*pUCP24-*floR-T1* and ΔPAO1*/*pUCP24-*floR-T1*) were only slightly increased in the presence of florfenicol or chloramphenicol.

### Characterization of the *floR* Variants

Using phylogenetic analysis, the amino acid identities of FloR-T1 and FloR-T2 with the known FloR proteins ranged from 90.80% to 100% and 86.10% to 88.90%, respectively (**Figure S1**). FloR-T1 was identical to the FloR protein (YP_001715371.1) identified in *Acinetobacter baumannii*, while FloR-T2 showed the highest identity (88.90%) with the FloR protein (YP_005351917.1) identified in *Klebsiella pneumoniae*.

**Figure 3:**
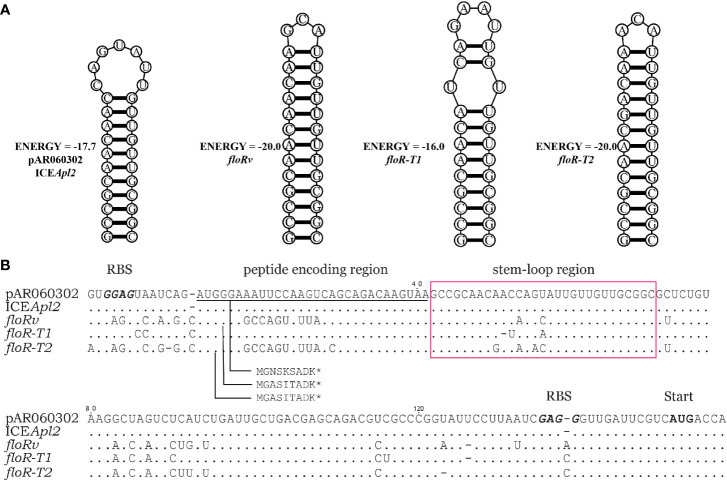
Comparison of sequences upstream of the *floR* variants. **(A)** The stable mRNA secondary structure of the *floR* variants formed by inverted repeat sequences boxed in **(B)**. **(B)** The attenuator of the *floR* variants consists of a peptide-encoding region (underlined) and stem-loop region (boxed). The start codons and ribosome binding sites (RBS) of the short peptide and *floR* are labeled and displayed in bold type letters.

The translational attenuator that consisted of a single pair inverted repeat (IR) sequence, and a short reading frame of 6-9 aa peptide was identified upstream of the *floR* variants (**Figure 3**). IR1 and IR2 can form a stable stem-loop structure blocking the resistance gene-associated ribosome binding site (RBS). The short peptides of *floR-T1* and *floR-T2* differ in three amino acids. The attenuator sequences of *floR-T2* and *floRv* encode an identical peptide, although one synonymous variation (A>T) in their nucleotide sequences. The attenuators’ IR resulted also differently, and the stem-loop structures formed in distinct stable states. Among these variants, *floR-T2* and *floRv* showed the most stable structure. However, the stable stem-loop structure of the attenuator sequence did not overlap with the RBS site of the *floR* gene.

### *floR-T2* Encoded in a Tn*4371*-Like ICE

**Figure 4:**
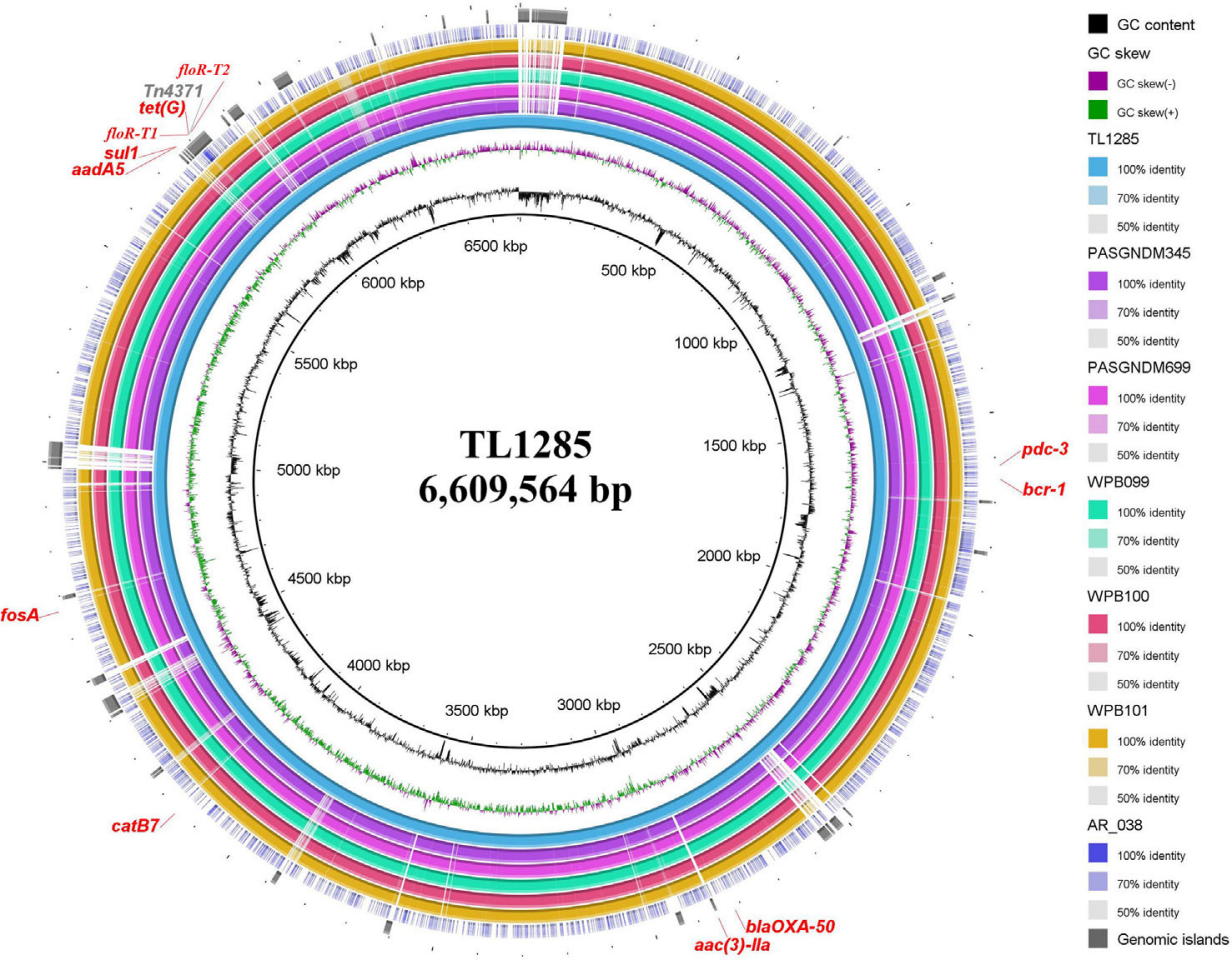
Sequence conservation among *P. aeruginosa* TL1285 and 6 other genomes carrying *floR-T2*. From innermost to outermost: Circle 1 shows the scale in kb; Circles 2 and 3 represent the GC content and GC skew maps of TL1285, respectively; Circle 4 represents the genome of TL1285; Circles 5-10 represent the homologous regions of PASGNDM345, PASGNDM699, WPB099, WPB100, WPB101 and AR_038 compared to those of TL1285, while the regions without similar hits between them were left blank; Circle 11 displays the genomic islands in TL1285; Circle 12 displays the antibiotic resistance genes in TL1285.

Whole genome sequencing (WGS) was performed for *P. aeruginosa* TL1285 carrying both *floR-T1* and *floR-T2*, and only produced a circular 6,609,407 bp chromosome with an average GC content of 66.06% encoding 5,611 ORFs. Multiple ARGs, including resistance genes for β-lactams (*bla*_OXA-50_ and *bla*_PDC-3_), aminoglycosides (*aadA5* and *aac(3)-IIa*), sulfonamides (*sul1*), tetracycline (*tetG*), chloramphenicol (*catB7*, *floR-T1* and *floR-T2*) and fosfomycin (*fosA*), were identified in the *P. aeruginosa* TL1285 genome. The florfenicol-resistant genes *floR-T1* and *floR-T2* were embedded in an 86-kb Tn*4371*-like integrative and conjugative element (ICE) (**Figure 4**).

To track the epidemiological correlation between *floR-T2* and genome islands, a BLASTN search was performed against the GenBank database using *floR-T2* as a query. A total of five *P. aeruginosa* chromosomes, WPB099 (CP031878), WPB100 (CP031877), WPB101 (CP031876), PASGNDM345 (CP020703) and PASGNDM699 (CP020704), and one *E. cloacae* chromosome, AR_038 (CP030347), were found carrying *floR-T2*. Through MLST analysis, the five *P. aeruginosa* belonged to ST308, while TL1285 to ST316. Interestingly, these *floR-T2-*carrying strains came from different sources. WPB099, WPB100 and WPB101 were isolated from hospital wastewaters in Singapore, PASGNDM345 and PASGNDM699 from patient sputum in Singapore, while *E. cloacae* AR_038 and TL1285 were from patient sputum collected in United States and China, respectively. Whole genome alignment of the six *P. aeruginosa* strains revealed high identity, and their differences were mainly in some genomic islands (**Figure 4**). The Tn*4371*-like ICE carrying *floR-T2* in TL1285 was also partially present in these five *P. aeruginosa* strains. Nevertheless, it should be noted that WPB099, WPB100 and WPB101 were not fully sequenced, and the *floR-T2* gene was located on an approximately 10 kb separate segment, which means the precise genetic environments around *floR-T2* could not be described.

**Figure 5:**
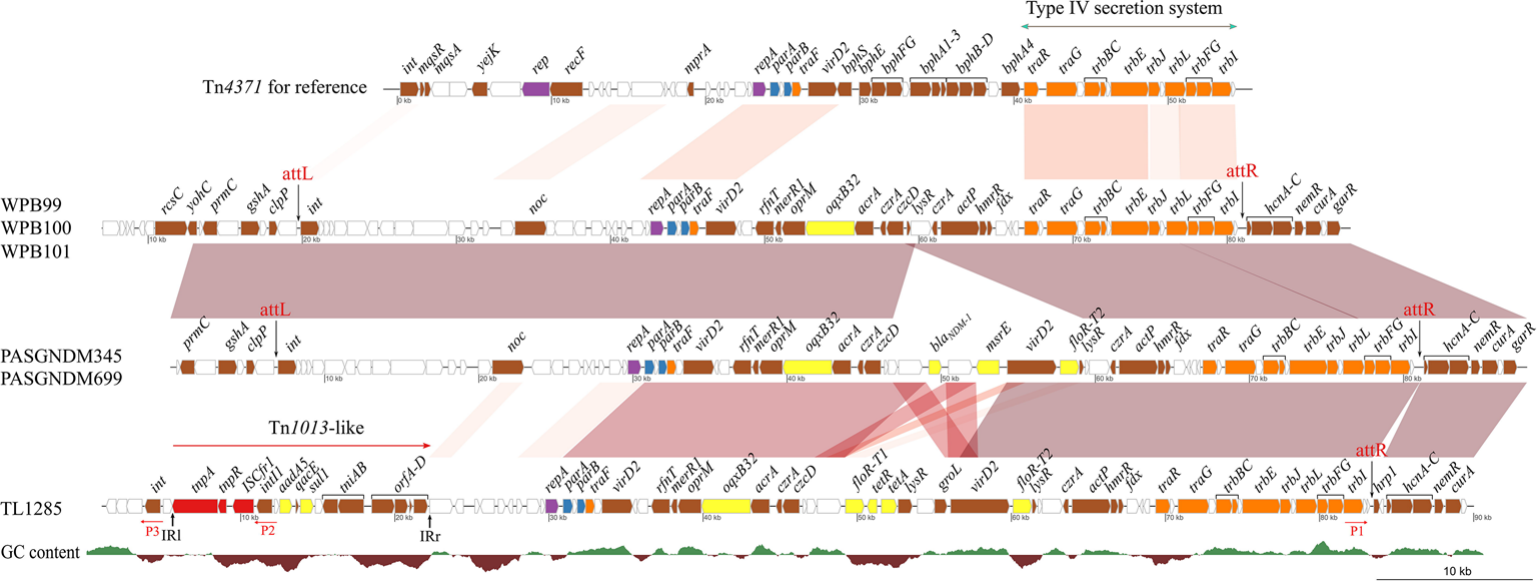
Comparative genomic analysis of the ICE region of TL1285 and 5 other *floR-T2*-carrying *P. aeruginosa* isolates. Genes with different functions are shown in different colors: red, transposable elements; yellow, drug resistance; orange, conjugational transfer; blue, plasmid maintenance; purple, replication; brown, genes with other functions; white, hypothetical proteins.

Comparative analysis of the Tn*4371*-like ICE regions of six *P. aeruginosa* strains revealed that the plasmid maintenance system (*repA, parA* and *parB*) and conjugational transfer systems were conserved (**Figure 5**). The variable region between the *traF* and *traR* genes, which encoded a biphenyl catabolic *bph* gene cluster in Tn*4371* (AJ536756), was different in these six *P. aeruginosa* isolates. The variable regions of WPB099, WPB100 and WPB101 were a 20-kb fragment encoding the *oqxB32* gene, which confers resistance to quinolone. The variable regions of PASGNDM345 and PASGNDM699 shared high identity with those of WPB099, WPB100 and WPB101. The only difference was that in PASGNDM345 and PASGNDM699, a 13.7-kb fragment flanked by 695 bp direct repeats was inserted between *czcD* and *lysR*, which encode *bla*_NDM-1_, *msr(E)* and *floR-T2* genes. The variable region of TL1285 was similar to those of PASGNDM345 and PASGNDM699, except that the *bla*_NDM-1_ *-hp-msr(E)* genes of PASGNDM345 and PASGNDM699 were replaced by *floR-T1-tetR-tetA-lysR* in TL1285.

The integrase genes (*int*) of PASGNDM345, PASGNDM699, WPB99, WPB100 and WPB101 were identical and shared 78% identity with that of Tn*4371*. However, no homologue of *int*_Tn_ *_4371_* was found in TL1285 (**Figure 5**). Tn*4371* family ICEs could be integrated into the genome through an 8-bp *attB* site, generating direct repeat *attL* and *attR* element chromosomal junctions (Merlin et al., 1999). In PASGNDM345, PASGNDM699, WPB99, WPB100 and WPB101, 8-bp repeats (5′-TTTTTTGT-3′) were identified in both extremities of the ICE region. However, in TL1285, only *attR* was found (**Figure 5**). The *noc* gene upstream of *repA* in TL1285 was truncated by a novel Tn*402* family transposon. The transposon is formed by IS*Cfr1* and In*2* carrying a single *aadA5* cassette embedded downstream of the *tnpR* gene of Tn*1013*, and this Tn*402* family transposon was surrounded by 37-bp imperfect inverted repeats (**Figure S2**).

Inverse PCR using primers P1, P2 and P3 (**Figure 5**) was performed to detect whether the ICE in TL1285 could generate a circular extrachromosomal form, but no positive result was observed. Taken together, we speculate that the ICE in TL1285 is an incomplete member of the Tn*4371* family and may have lost the excising or integrating ability. The insertion of the Tn*402* family transposon leads to the loss of the upstream sequence of the nucleoid occlusion protein coding gene *noc*, including the integrase gene *int* of ICE.

## Discussion

In this work, we found that among the 430 clinical *P. aeruginosa* isolates detected, most (94.65%, 407/430) of them were resistant to florfenicol or/and chloramphenicol. In fact, *P. aeruginosa* was intrinsically resistant to amphenicols, and the MICs to florfenicol and chloramphenicol for *P. aeruginosa* ATCC27853 were both 64 μg/mL (Fass and Barnishan, 1979). Active efflux and chloramphenicol acetyltransferase (CAT) encoded on *P. aeruginosa* chromosome are two major mechanisms of amphenicol resistance (Nitzan and Rushansky, 1981; Li et al., 1994), and different types of CAT determinant also contribute to amphenicol resistance (White et al., 1999). However, there were still 5.35% (23/430) isolates susceptible to amphenicol. The mutation and deletion of multidrug efflux system (such as MexA-MexB-OprK) and other resistance mechanisms might play a role in the loss of resistance to florfenicol or/and chloramphenicol of these bacteria.

Three *floR* variants (*floR, floR-T1* and *floR-T2*) were identified in a number of clinical *P. aeruginosa* isolates, in which *floR-T1* was the most prevalent variant and *floR-T2* was a novel variant identified in this study. The positive rate of the *floR-T1* gene in the strains collected from 2015-2017 (7.39%, 17/230) was similar to that of the clinical *K. pneumoniae* isolates collected from the same district during 2010-2014 (7.01%, 23/328) (Lu et al., 2018). The protein showing the highest identity (88.90%) with FloR-T2 was a FloR protein (YP_005351917.1) identified in *Klebsiella pneumoniae*. Currently, FloRv was the FloR variant with the lowest identity (88.40%-91.80%) to other previously reported FloR proteins (He et al., 2015). FloR-T2 exhibited less identity with other FloR proteins than FloRv. Furthermore, FloR-T2 was shown to be one of the most divergent members of the FloR family, followed by FloRv (**Figure S1**).

It was interesting to find that the expression levels of *floR-T2* increased much more significantly in the host TL1285 or the recombinant than those of *floR-T1* when induced by the amphenicols. Using transcriptome sequencing, Lang et al. found that the expression of the *floR* gene of the *E. coli* plasmid pAR060302 increased 8-fold under the induction of florfenicol (Lang et al., 2012). Yinghui et al. also reported that the mRNA levels of the *floR* gene encoded by ICEApl2 on chromosomes increased in the presence of chloramphenicol (Li et al., 2018). However, the modulation mechanisms of mRNA expression of *floR* variants remain unclear. As reported by Yinghui et al. (Li et al., 2018), we also identified the translational attenuator region upstream of the *floR* variants. In addition, we found that the peptide encoding region of *floR-T1* was identical to those of pA060302 and ICEApl2 reported by Yinghui (Li et al., 2018) (**Figure 3**). It is known that the expression of chloramphenicol resistance genes, including *catA*, *cmlA* and *fexA*, could be induced by chloramphenicol, and this induction is mediated by translational attenuator structure at the post-transcriptional level (Stokes and Hall, 1991; Kehrenberg and Schwarz, 2004; Schwarz et al., 2004). However, considering that the stem-loop structure is distant to the RBS site of the *floR* gene, it is not clear whether this structure participates in the induced expression of the *floR* gene.

WGS result revealed that *floR-T1 and floR-T2* of *P. aeruginosa* TL1285 were related with a novel Tn*4371*-like ICE. Tn*4371* is a 55-kb ICE that can be integrated into the *attB* site (5’-TTTTCAT-3’) through a site-specific recombination process since the ends of the element can be detected covalently as a transfer intermediate (Merlin et al., 1999; Toussaint et al., 2003). The Tn*4371*-like ICEs are mosaic in structure and consist of Ti-RP4-like transfer systems, an integrase region, plasmid maintenance genes and accessory genes (Toussaint et al., 2003). Any ICE that encodes an integrase gene closely related to *int*_Tn_ *_4371_* (>70% protein homology) and has similar maintenance and transfer genes could be considered as a member of the Tn*4371* family (Ryan et al., 2009). The Tn*4371*-like ICEs carrying *floR* variants have been identified in the *P. aeruginosa* strains of different MLST types (such as ST308 and ST316) isolated from different samples of different countries. *P. aeruginosa* ST308 is a high-risk clone that can locally acquire resistance determinants from water-distribution system and was involved in a five-year outbreak in a French hospital between 2005 and 2010 (Jeanvoine et al., 2019). The variable region of these Tn*4371*-like ICEs also carried other ARGs like *bla*_NDM-1_, *tetA* and *msr(E)*. These findings indicate that the Tn*4371*-like ICEs might have emerged as a potential vehicle to mediate the spread of drug resistance genes in *P. aeruginosa* isolates.

## Conclusion

In this study, we determined the prevalence of *floR* among 430 clinical isolates of *P. aeruginosa* and characterized two *floR* variants, *floR-T1* and *floR-T2*, in a *P. aeruginosa* strain TL1285. The *floR-T1* gene was the most prevalent variant in clinical *P. aeruginosa* strains. The *floR-T2* is a novel *floR* variant that showed less identities with the other FloR proteins than FloRv. The mRNA levels of the two *floR* variants could be induced by florfenicol and chloramphenicol and the expression level of *floR-T2* was significantly higher than that of *floR*-T1. Inverted repeat sequences as well as stem-loop regions of the translational attenuators differed among the *floR* variants. The *floR-T1* and *floR-T2* of TL1285 were located on an incomplete novel Tn*4371* family ICE, while *floR-T2*-carrying ICEs were also identified in other five *P. aeruginosa* genomes. These results indicate that Tn*4371* family ICEs might be related with the dissemination of *floR-T2* among *P. aeruginosa* strains.

## Data Availability Statement

The datasets presented in this study can be found in online repositories. The names of the repository/repositories and accession number(s) can be found in the article/**Supplementary Material**.

## Ethics Statement

Ethical review and approval was not required for the study on human participants in accordance with the local legislation and institutional requirements. Written informed consent for participation was not required for this study in accordance with the national legislation and the institutional requirements.

## Author Contributions

JL, HX, HYL, XL, KL, and HZ collected the strains. HML, WL, JC, XZhang, KS, and QL performed the experiments. QC, HLL, and XZhu analyzed the experimental results. CQ, WZ, and AL performed the bioinformatics analysis. CQ, TX, and QB co-led the writing of the manuscript. HZ, ZM, and QB designed the work. All authors contributed to the article and approved the submitted version.

## Funding

This study was supported by the Science and Technology Project of Wenzhou City, China (Y20170205 and 2019Y0358), Science and Technology Project of Lishui City, China (2017GYX07), the National Natural Science Foundation of China (81973382, 81960381), the Natural Science Foundation of Zhejiang Province, China (LQ17H190001 and LY19C060002) and the Science & Technology Project of Inner Mongolia Autonomous Region, China (201802125).

## Conflict of Interest

The authors declare that the research was conducted in the absence of any commercial or financial relationships that could be construed as potential conflict of interest.
