# Supplementary material for: Identification of floR Variants Associated With a Novel Tn4371-Like Integrative and Conjugative Element in Clinical Pseudomonas aeruginosa Isolates
Source: Front Cell Infect Microbiol. 2021 Jun 21;11:685068. doi: 10.3389/fcimb.2021.685068 (PMC8256890; doi:10.3389/fcimb.2021.685068)
Supplement: Supplementary file 1 [file DataSheet_1.pdf]

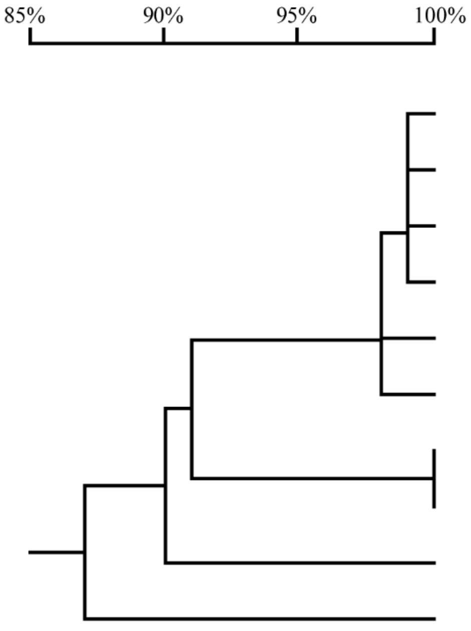

Homology matrix

P.multocida FloR [EJS85950.1]	100%									
V.cholerae FloR [AAK64587.1]	99.30%	100%								
A.bestiarum FloR [YP_001220605.1]	99.00%	98.80%	100%							
E.coli FloR [AAS16362.1]	99.00%	98.80%	98.50%	100%						
S.enterica Typhimurium FloR [YP_008672309.1]	98.50%	98.30%	98.30%	98.00%	100%					
K.pneumoniae FloR [YP_005351917.1]	98.00%	97.80%	97.50%	97.50%	97.00%	100%				
A.baumannii FloR [YP_001715371.1]	91.30%	91.10%	91.10%	90.80%	92.80%	90.80%	100%			
FloR_T1	91.30%	91.10%	91.10%	90.80%	92.80%	90.80%	100.00%	100%		
S.maltophilia FloRv [AIU94575.1]	89.90%	89.60%	89.60%	89.40%	91.30%	91.80%	88.40%	88.40%	100%	
FloR_T2	87.60%	87.40%	87.40%	87.10%	88.40%	88.90%	86.10%	86.10%	88.40%	100%

Figure S1. Homology tree of functional FloR proteins and homology matrix of these protein sequences.

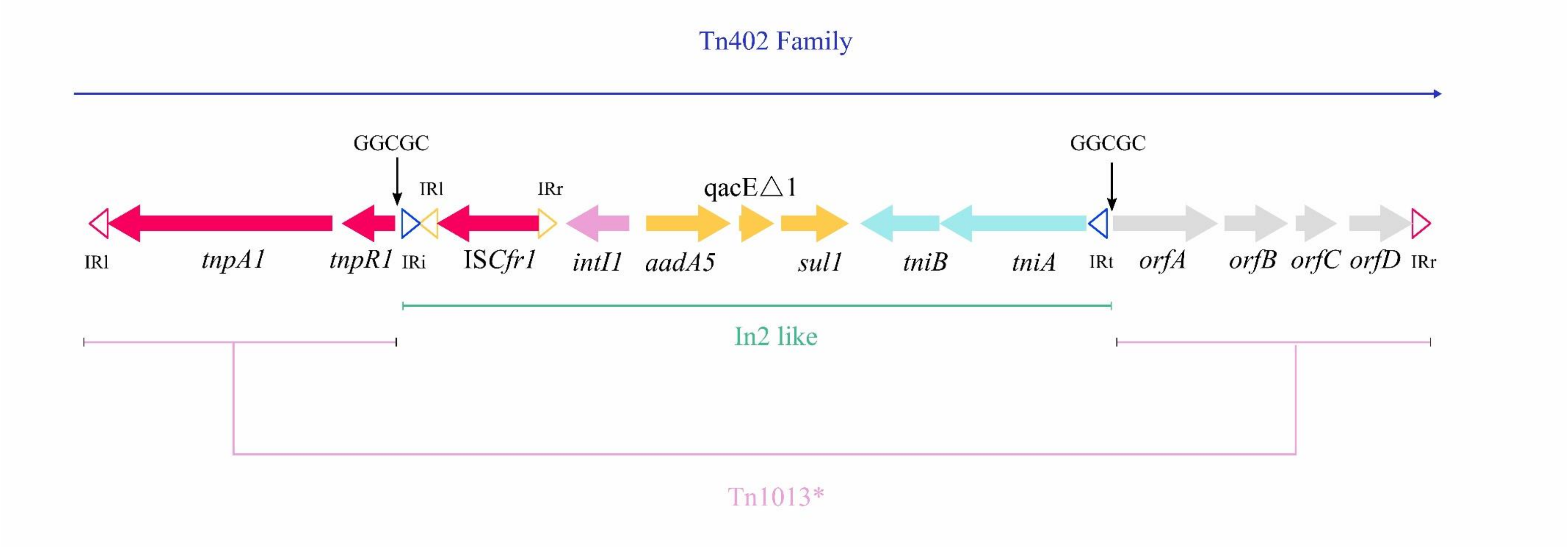

Figure S2. Structure of the Tn402 family transposon of TL1285.
